# Cigarette Smoke Extract Activates Hypoxia-Inducible Factors in a Reactive Oxygen Species-Dependent Manner in Stroma Cells from Human Endometrium

**DOI:** 10.3390/antiox10010048

**Published:** 2021-01-03

**Authors:** Naoko Kida, Yoshiyuki Matsuo, Yoshiko Hashimoto, Kenichiro Nishi, Tomoko Tsuzuki-Nakao, Hidemasa Bono, Tetsuo Maruyama, Kiichi Hirota, Hidetaka Okada

**Affiliations:** 1 Department of Obstetrics and Gynecology, Kansai Medical University, 2-3-1 shinmachi-cho, Hirakata 573-1191, Japan; kidanao@hirakata.kmu.ac.jp (N.K.); hashiyos@hirakata.kmu.ac.jp (Y.H.); tsuzukto@hirakata.kmu.ac.jp (T.T.-N.); hokada@hirakata.kmu.ac.jp (H.O.); 2 Department of Human Stress Response Science, Institute of Biomedical Science, Kansai Medical University, Hirakata 573-1010, Japan; ysmatsuo-kyt@umin.ac.jp (Y.M.); nishik@hirakata.kmu.ac.jp (K.N.); 3 Graduate School of Integrated Sciences for Life, Hiroshima University, Higashi-Hiroshima City 739-0046, Japan; bonohu@hiroshima-u.ac.jp; 4 Department of Obstetrics and Gynecology, Keio University School of Medicine, Tokyo 160‐8582, Japan; tetsuo@keio.jp

**Keywords:** cigarette smoking, hypoxia-inducible factor, HIF, CS extract, endometrial stromal cell, reactive oxygen species, immortalized cell line

## Abstract

Cigarette smoking (CS) is a major contributing factor in the development of a large number of fatal and debilitating disorders, including degenerative diseases and cancers. Smoking and passive smoking also affect the establishment and maintenance of pregnancy. However, to the best of our knowledge, the effects of smoking on the human endometrium remain poorly understood. In this study, we investigated the regulatory mechanism underlying CS-induced hypoxia-inducible factor (HIF)-1α activation using primary human endometrial stromal cells and an immortalized cell line (KC02-44D). We found that the CS extract (CSE) increased reactive oxygen species levels and stimulated HIF-1α protein stabilization in endometrial stromal cells, and that CS-induced HIF-1α-dependent gene expression under non-hypoxic conditions in a concentration- and time-dependent manner. Additionally, we revealed the upregulated expression of a hypoxia-induced gene set following the CSE treatment, even under normoxic conditions. These results indicated that HIF-1α might play an important role in CS-exposure-induced cellular stress, inflammation, and endometrial remodeling.

## 1. Introduction

Cigarette smoking (CS) is a major contributing factor in the development of a large number of fatal and debilitating disorders, including degenerative diseases and cancers [1,2,3,4,5]. Additionally, smoking and passive smoking affect the establishment and maintenance of pregnancy. The association between tobacco consumption and female infertility in natural cycles has been consistently reported in epidemiological studies [6,7,8,9]. Moreover, the effect of smoking on fetal factors reportedly shows deleterious changes in the placenta and fetus. It is more frequently observed in pregnant smokers presenting higher rates of low birth weight and perinatal and neonatal mortality [2,10,11]. However, to the best of our knowledge, the effects of smoking on the human endometrium remain poorly understood.

Implantation describes the series of processes from embryo adhesion to the endometrium, invasion of the embryo into the endometrium, and formation of the placenta, and represents the phenomenon that marks the beginning of pregnancy. Previous reports showed that oxygen concentration on the surface of the endometrium at the beginning of human pregnancy is lower than that inside of the endometrium [12,13]. These results suggest that the endometrium, especially during decidualization, is less oxygenated.

The transcription factor hypoxia-inducible factor (HIF), which plays an essential role in cellular response to changes in oxygen partial pressure in the intrauterine environment (where oxygen metabolism is highly variable), plays an important role in major cellular responses to hypoxia and regulation of oxygen homeostasis [14]. Previous studies have examined the significance of hypoxia in placentation and the period of hyperoxia following implantation [15,16]. HIF is a major transcription factor that responds to low oxygen tension and induces the expression of hypoxia-related genes, such as vascular endothelial growth factor (*VEGF*), induction of which affect various physiological responses, such as metabolism, cell death, and angiogenesis. HIF comprises two subunits (α and β), and the HIFα subunit has three isotypes (HIF-1α, HIF-2α, and HIF-3α) [17]. A previous study showed that the expression of *Hif1a* is primarily found in the uterine luminal epithelium during the peri-implantation period, and that *Hif2a* expression is mainly found in the stroma at the same time, whereas *Hif3a* expression is undetectable in the uterus [18]. Notably, *Hif2a* is strongly expressed in the uterine stroma after the embryo attachment [15]. These findings suggest that HIFα plays functional roles in embryo implantation.

HIF activation is regulated not only by changes in oxygen partial pressure but also various other external factors, including intercellular signaling molecules, such as nitric oxide, proinflammatory cytokines, and inflammatory substances, including lipopolysaccharide [14,19]. One such factor is tobacco, with one study showing that cigarette smoke extract (CSE) increases the HIF-1 activity in cells under normoxic partial pressure [20]. However, few studies have focused on the hypoxic environment of the endometrium prior to implantation, and the implications of CS in this environment of the endometrium remain largely unknown. We previously demonstrated that CS and treatment with CSE induce HIF-1 activation in alveolar and bronchial epithelium-derived cells [20].

This study’s primary endpoint was to determine whether CSE activates transcription factors HIFs, including HIF-1 and HIF-2, in endometrial stromal cells and in immortalized cells that we established from stromal-derived cells. For these purposes, we quantified the proteins of HIF subunits, such as HIF-1α, HIF-2α, and HIF-1β, and examined the concentration- and time- dependency of CSE effects. To comprehensively explore the biological significance of HIF activation, we performed RNA-Seq assays using next-generation sequencers to investigate the similarities and differences in the pathways of gene expression regulation by CSE and HIF activation. Moreover, we investigated the intracellular signal transduction of CSE-induced HIF activation. These results indicated that HIF-1α might play an important role in CS-exposure-induced cellular stress, inflammation, and remodeling of the endometrium.

## 2. Materials and Methods

### 2.1. Reagents

Reagents and materials used in this study are listed as Table A1.

### 2.2. Tissue Collection

Human tissues were obtained with written informed consent from each patient in accordance with the Declaration of Helsinki. Human endometrial tissue in the proliferative phase were obtained from two women who underwent hysterectomies due to uterine fibroids. The subjects had regular menstrual cycles and no preoperative hormonal treatment. A section of each endometrial specimen was analyzed and confirmed as histologcally normal. This study was approved by the institutional review board of Kansai Medical University (Osaka, Japan; project approval no. 2006101) [21,22].

### 2.3. Cell Culture

Human endometrial stromal cells (ESCs) were purified from endometrial tissues using the standard enzyme-digestion method previously described [21,22,23]. ESCs were cultured in Dulbecco’s modified Eagle medium (DMEM)/F-12 supplemented with 10% fetal calf serum (FCS; Sigma-Aldrich, St Louis, MO, USA), 100 IU/mL penicillin, 100 mg/mL streptomycin, and 0.25 μg/mL amphotericin B (antibiotic-antimycotic 100×; Gibco, Gaithersburg, MD, USA) at 37 °C in a humidified atmosphere of 5% CO_2_. After subsequent pipetting, the cell suspension was diluted with a 2-volume DMEM/F-12 medium and placed in a centrifugation tube (Corning Glass Works, Corning, NY, USA), where it remained upright for 10 min at unit gravity. The supernatant, excluding the lowermost 2 mL, was transferred into a new tube to collect the suspended single cells. After repeating this procedure several times, the cell suspension was washed three times and used as a source of ESC. The viability, determined by dye exclusion, was ≥90%. Two million viable ESCs were cultured in 75 cm^2^ flasks in the DMEM/F-12 medium supplemented with 10% fetal calf serum (FCS; HyClone, Logan, UT, USA), 100 IU/mL penicillin, and 100 μg/mL streptomycin (Gibco) at 37 °C in a humidified atmosphere of 5% CO_2_ in air. The culture medium was replaced 60 min after plating to reduce epithelial cell contamination. The percentage of vimentin-positive cells among 80%-confluent ESCs was confirmed between 96 and 99% according to immunohistochemical staining, as previously described [21,22,23]. After passage 0 to 1 when ESCs were nearly confluent, the cells were trypsinized and replated. To negate the effect of endogenous steroid hormones, cells were cultured until confluence, and the medium was replaced with Phenol Red-free DMEM/F-12 supplemented with 10% dextran-coated charcoal stripped (DCS)-FCS, antibiotic-antimycotic 1× (Gibco), and 2 mM L-alanyl-L-glutamine (GlutaMAX; Gibco). After 48 h, ESCs were cultured in DCS-FCS. ESCs were treated with CSE diluted by culture media at various concentrations (0.01, 0.025, 0.1, and 0.25%) or untreated, in addition to treatment with ovarian sex-steroid hormones.

The hTERT-immortalized ESC line KC02-44D hTERT was purchased from ATCC (ATCC SC-6000; Manassas, VA, USA) [24] and maintained in DMEM supplemented with 10% fetal bovine serum, 100 U/mL penicillin, 100 mg/mL streptomycin, and 2 mg/mL blasticidine at 37 °C under 5% CO_2_ in a humidified incubator. KC02-44D, by introducing the hTERT gene into primary ESCs showed characteristics such as decidualization capacity and responses to IL-1β similar to primary human ESCs. It is reported that the KC02-44D cell line is useful to investigate the mechanisms of normal and pathological human reproductive processes [24].

### 2.4. Preparation of CSE

A detailed protocol for CSE preparation is available at protocols.io (https://dx.doi.org/10.17504/protocols.io.bnymmfu6). Briefly, smoke from five cigarettes (Mevius original; 10 mg tar, 0.8 mg nicotine; Japan Tobacco Inc., Tokyo, Japan) was bubbled through 10 mL of phosphate-buffered saline using a syringe pump at a flow rate of 300 mL/min. The extract was filtered through a 0.2-µm pore-size membrane, and the resulting solution was defined as 100% CSE. Previous studies report that 1% CSE can be derived from fewer than two packs of cigarettes per day [25,26], therefore, we used 0.5 to 4% CSE for this study.

### 2.5. Cell Proliferation Assay

The proliferation rate of ESCs was detected using a Cell Counting Kit-8 (CCK-8; Dojindo, Shanghai, China) according to the manufacturer’s instructions. Briefly, ESCs were seeded in 96-well plates at a density of 5 × 10^3^ cells/well, and after culturing for 24 h, treated with increasing concentrations of CSE (0, 1, 2, or 4 %) for 24 h. The CCK-8 stain was subsequently added, and the optical density at 450 nm was detected using a microplate reader (EnSpire; PerkinElmer, Inc., Billerica, MA, USA). Each experiment was repeated six times.

### 2.6. Western Blot Analysis

Whole-cell lysates were prepared using a lysis buffer containing the mammalian protein-extraction reagent (Thermo Fisher Scientific, Rockford, IL, USA) and a protease-inhibitor cocktail (Calbiochem, La Jolla, CA, USA). Samples were centrifuged at 10,000× *g* to pellet the cell debris. Protein concentrations were quantified using the Bio-Rad protein assay reagent (Bio-Rad, Hercules, CA, USA). Equivalent amounts of lysate protein (20 μg/lane) were electrophoresed on a 7.5% sodium dodecyl sulfate polyacrylamide gel and electro-transferred onto membranes using the Trans-Blot Turbo transfer system (Bio-Rad). Nonspecific binding sites were blocked with the Blocking One solution (Nacalai Tesque, Kyoto, Japan) for 20 min. The membranes were probed with purified mouse anti-human HIF-1α (1:1000; BD Transduction Laboratories, Tokyo, Japan), rabbit polyclonal anti-HIF-2α (1:1000; Novus Biologicals, Centennial CO, USA), rabbit monoclonal anti-HIF-1β (1:1000; Cell Signaling Technology, Danvers, MA, USA), and mouse monoclonal anti-β-actin (1:5000; Sigma-Aldrich) as the primary antibodies and anti-mouse IgG peroxidase-labeled secondary antibody (1:5000; GE Healthcare Life Science, Pittsburgh, PA, USA) as the secondary antibody. Immune complexes were visualized using enhanced chemiluminescence plus Western blotting detection reagents (GE Healthcare Life Science). Experiments were performed in triplicate, and representative blots are shown.

### 2.7. ROS Assay

The ROS-Glo^TM^ H_2_O_2_ assay was used to measure ROS in the homogeneous assay (lytic) mode. Cells were seeded in 96-well plates and treated with CSE for 4 h, followed by the addition of H_2_O_2_ substrate-dilution buffer and H_2_O_2_ substrate. After 20 min, luminescence units were measured using a microplate reader (EnSpire; PerkinElmer, Inc., Billerica, MA, USA). For each experiment, cells were assayed in triplicates for each condition.

### 2.8. Luciferase Assay

The reporter plasmid pGL3/5HRE-luc (HeLa/5HRE-luc) [27], the pGL3/ODD-luc plasmid [28], and pGL3/HIF-1α 5′FS-luc [29] were kindly provided by Dr. Hiroshi Harada (Kyoto University, Kyoto, Japan). KC02-44D cells were transfected with each plasmid using the *Trans*IT-LT1 transfection reagent (Mirus Bio LLC, Madison, WI, USA), and cell lysates were subjected to luciferase assays using the ONE-Glo luciferase assay system (Promega, Madison). For each experiment, at least two independent transfections were assayed in triplicates.

### 2.9. Semi-Quantitative Reverse Transcription Polymerase Chain Reaction (RT-PCR)

Total RNA was isolated from cells using the RNeasy Mini kit (Qiagen, Hilden, Germany) according to the manufacturer’s instructions. The first-strand cDNA synthesis kit ReverTra Ace qPCR RT master mix (TOYOBO, Osaka, Japan) was used for cDNA synthesis, and reverse transcription was performed according to the manufacturer’s instructions. Real-time PCR was performed using the Rotor-Gene Q HRM (Qiagen) and the Thunderbird SYBR qPCR mix kit (TOYOBO) according to the manufacturer’s instructions. The relative gene expression was calculated using the delta-delta Ct method. Ct values were normalized against the elongation factor-1α used as an internal control. The primer sequences are listed in Table 1 [30].

### 2.10. RNA-Seq

Total RNA was extracted from cells using an RNeasy Mini kit (Qiagen) and processed using a TruSeq Stranded mRNA sample prep kit (Illumina, San Diego, CA, USA) [31]. Poly(A) RNA libraries were then constructed using a TruSeq Stranded mRNA library preparation kit (Illumina) and sequenced in 100-bp paired-end reads on an Illumina NovaSeq6000 platform [31]. Sequencing data were deposited into the DNA Data Bank of Japan Sequence Read Archive (accession nos. DRR228416–DRR228423). RNA-Seq was performed in duplicates.

### 2.11. Transcriptomics Analysis

RNA-Seq reads were quantified using ikra (v.1.2.2) [32], an RNA-Seq pipeline centered on Salmon [33]. Ikra automated the RNA-Seq data-analysis process, including the quality control of reads [sra-tools v.2.10.7, Trim Galore v.0.6.3 [34] using Cutadapt v.1.9.1 [35], and transcript quantification (Salmon v.0.14.0, using reference transcript sets in GENCODE release 31 for humans), and tximport v.1.6.0). These tools were used with default parameters. Count tables were imported into integrated differential expression and pathway analysis (iDEP v.0.91), an integrated web application for gene ontology (GO) analysis of RNA-Seq data [36]. Quantified transcript reads were filtered at a threshold of 0.5 counts per million (CPM) in at least one sample and transformed using EdgeR (3.28.0): log2(CPM + c), with a pseudocount of 4. The gene set enrichment analysis (GSEA) was performed in iDEP using fold-change values returned by DESeq2 (1.26.0). False positive rates of *q* < 0.05 were considered enriched and investigated further by Metascape [37]. The TRRUST method [38,39], which analyzes human transcriptional regulatory interactions, was applied using the web-based Metascape [37] algorithm.

### 2.12. Statistics

Data are presented as the mean ± SD when indicated in figure legends. Differences between groups were evaluated with the one-way analysis of variance (ANOVA) and two-way ANOVA followed by Dunnett’s test for multiple comparisons. Statistical analyses were performed using GraphPad Prism 8 (GraphPad Software, Inc. La Jolla, CA, USA). Statistical significance was defined as *p* < 0.05.

## 3. Results

### 3.1. CSE Induces HIF-1α Protein Accumulation under Non-Hypoxic Conditions in Primary Cultured Human ESCs

**Figure 1:**
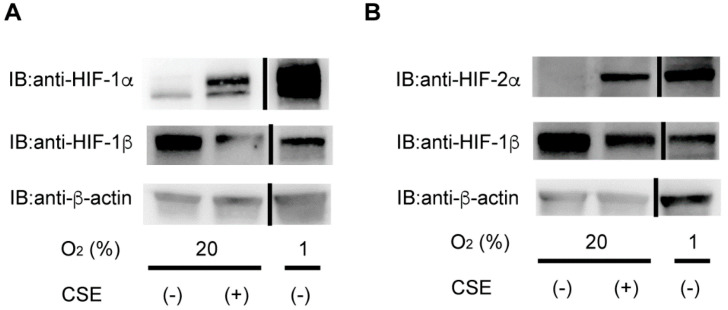
Cigarette smoke extract (CSE) induces HIF-1α protein accumulation under non-hypoxic conditions in primary cultured human endometrial stromal cells (ESCs). (**A**,**B**) Primary cultured human ESCs were exposed to culture media (-), 2% CSE (+) or 1% O_2_ for 4 h, followed by harvesting and immunoblotting of whole-cell lysates for HIF-1α (**A**), HIF-2α (**B**), HIF-1β, and β-actin. Experiments were performed at least twice, and representative immunoblots are shown.

To examine the effect of CSE on HIF-1 and HIF-2, human ESCs were exposed to 2% CSE under 20% O_2_ or 1% O_2_ for 4 h, due to the fact that our preliminary experiments indicated that 4 h is the most efficient period for CSE to induce HIF-1α protein. Exposure to 1% O_2_ resulted in upregulated levels of HIF-1α protein, and CSE exposure also induced HIF-1α protein expression within 4 h, although at levels lower than those induced by 1% O_2_ (Figure 1A).

The HIF-2α protein level was also upregulated by 2% CSE as well as by exposure to 1% O_2_ (Figure 1B). HIF-1β and β-actin levels were unaffected by the CSE treatment or 1% O_2_.

### 3.2. CSE Induces HIF-1α Protein Accumulation under Non-Hypoxic Conditions in Immortalized KC02-44D Cells 

**Figure 2:**
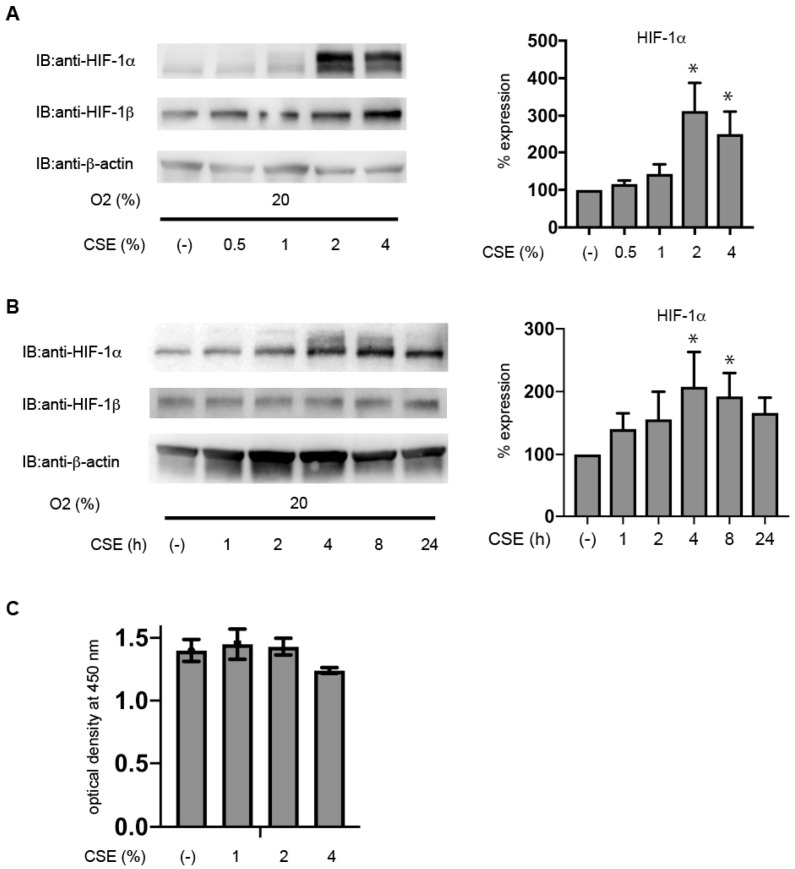
CSE induces HIF-1α protein accumulation under non-hypoxic conditions in immortalized KC02-44D cells. (**A**,**B**) Immortalized KC02-44D cells were exposed to the indicated concentrations of (**A**) or 2% (**B**) CSE for 4 h (**A**) and the indicated times (**B**). After the treatment, cells were harvested, and whole-cell lysates were immunoblotted for HIF-1α, HIF-1β, and β-actin. Representative immunoblots are shown. The right panel shows the results of densitometric analysis normalized by β-actin. Data represent the mean ± standard deviation (SD) (*n* = 3). * *p* < 0.05 compared to the control (CSE(-)). (**C**) KC02-44D cells were cultured for 24 h in a medium containing the indicated concentration of CSE (1–4%) or no treatment (control). Cells were subsequently incubated with a CCK-8 solution for 2 h, and absorbance was measured at 450 nm. Data represent the mean ± SD (*n* = 5).

Immortalized KC02-44D cells were exposed to 0.5, 1, 2, or 4% CSE under 20% O_2_ for 4 h. We found that >1% CSE significantly induced HIF-1α protein expression, even under 20% O_2_ conditions (Figure 2A). CSE effects peaked at 4 h, followed by a gradual decline in HIF-1α protein levels by 24 h (Figure 2B), suggesting a dose- and time-dependent effect. Additionally, HIF-1β or β-actin levels were unaffected by the CSE treatment or 1% O_2_, and the CSE treatment did not affect cell proliferation (Figure 2C). These data showed that the CSE concentration used in the present study was not significantly toxic to KC02-44D cells.

### 3.3. Global Gene-Expression Analysis Using RNA-Seq and Next-Generation Sequencing (NGS)

**Figure 3:**
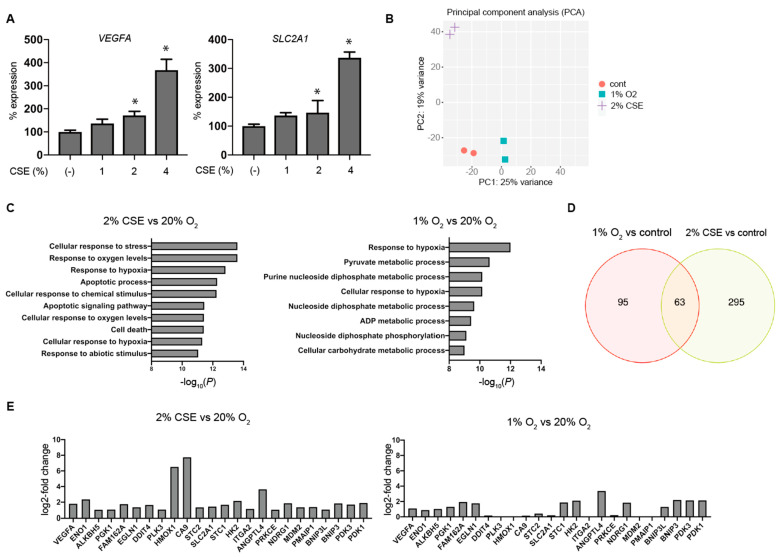
Global gene-expression analysis by RNA-Seq analysis using next-generation sequencing (NGS). KC02-44D cells were exposed to the indicated concentrations of CSE for 8 h under 20% O_2_ conditions, and total RNAs were harvested and (**A**) analyzed by semi-quantitative RT-PCR to assess VEGFA and SLC2A1. Data represent the mean ± SD (*n* = 3). * *p* < 0.05 vs. control. (**B–E**) Total RNAs were analyzed by RNA-Seq. (**B**) PCA of the RNA-Seq data. (**C**) GSEA, with the gene sets separated according to the GO term. (**D**) Venn diagram of genes showing significantly altered expression. (**E**) Changes in the expression levels of selected genes within the GO term “cellular response to hypoxia” (GO:0071456).

**Figure 4:**
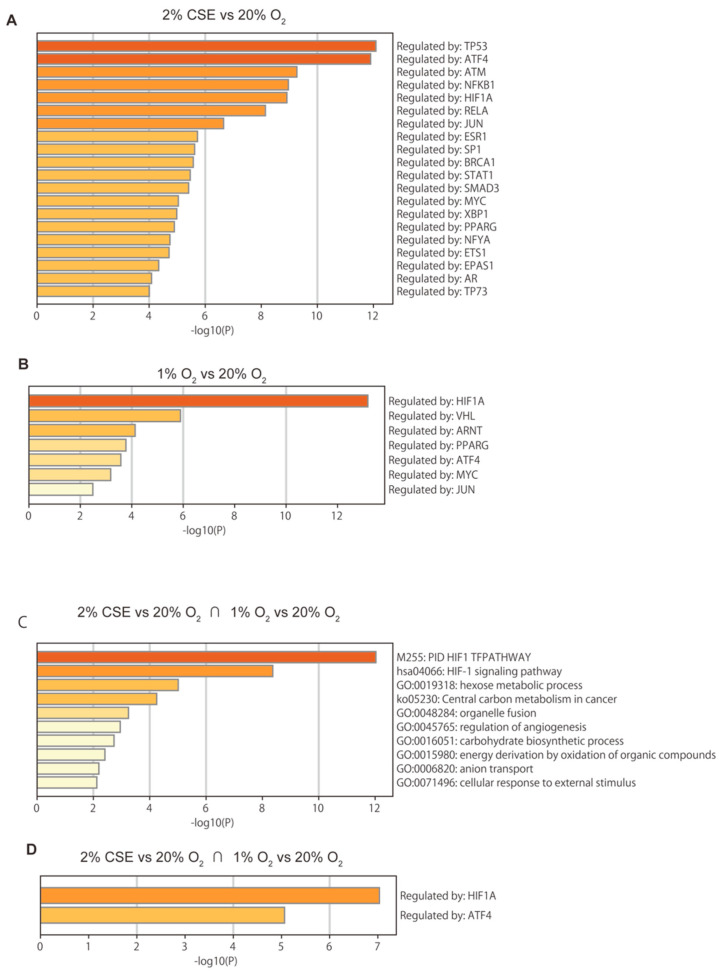
Gene set enrichment analysis (GSEA) (**A–D**) for each gene list, pathway and process-enrichment analysis were performed using TRRUST, a reference database of human transcriptional regulatory interactions (**A**,**B**,**D**) and GSEA according to the gene ontology (GO) term (**C**). Terms with a *p* < 0.01, a minimum count of 3, and an enrichment factor >1.5 (the ratio of observed counts to those expected by chance) were collected and grouped into clusters based on their membership similarities. The *p*-values were calculated based on the accumulative hypergeometric distribution, and *q*-values were calculated using the Banjamini–Hochberg procedure to account for multiple testing. The most statistically significant term within a cluster was chosen to represent the cluster.

An evaluation of mRNA levels of VEGFA and SLC2A1 (encoding glucose transporter 1) by semi-quantitative real-time RT-PCR indicated that the treatment with 1 to 4% CSE increased both levels within 8 h (Figure 3A). Additionally, we assessed genome-wide gene-expression patterns via RNA-Seq using NGS to determine the effects of CSE and hypoxia (1% O_2_ conditions). The expression matrix calculated as scaled transcripts per kilobase million was analyzed by iDEP.91 for differential expression and pathway analysis, and principal component analysis (PCA) was performed on the RNA-Seq data (Figure 3B). The gene set enrichment analysis (GSEA) of the intersection of the two gene lists revealed typical features of hypoxia-inducible genes (Figure 3C), and genes with significant changes in the expression ratio under exposure to 1% O_2_ and 2% CSE were compared to identify the differences (Figure 3D). A common gene list was then subjected to another round of GSEA. RNA-Seq identified CSE-mediated differences in the expression levels of selected genes within GO:0071456 (cellular response to hypoxia) under normoxic conditions, suggesting that CSE induced a genome-wide change in gene expression similar to the change elicited by the hypoxia treatment (Figure 3E). We then used TRRUST to examine which transcription-factor-dependent gene groups were enriched [38,39] (Figure 4A–D). For 1% O_2_ stimulation, HIF1A was highly enriched, whereas for the CSE treatment, TP53, ATM, and NFKB1 ranked higher than HIF1A (Figure 4A). This finding suggested that although tobacco strongly activates hypoxia-dependent pathways, it appears to drive the expression of a more complex group of genes through other signaling pathways. Furthermore, we identified a common gene cluster between hypoxia and CSE driven by HIF1A and ATF4 (Figure 4D).

### 3.4. CSE Selectively Stabilizes HIF-1α Levels rather than HIF-2α

**Figure 5:**
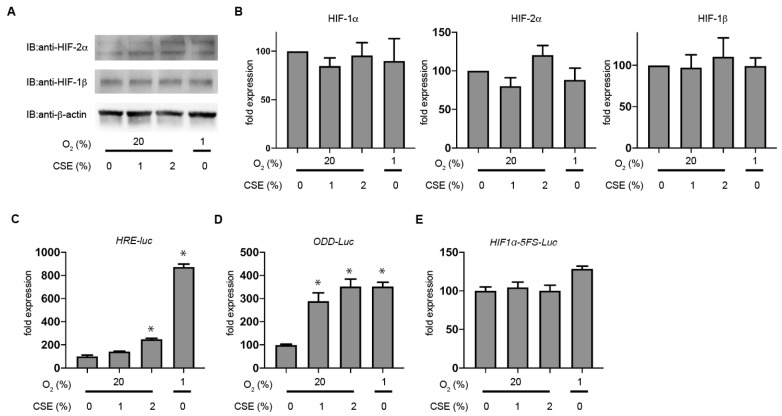
CSE selectively stabilizes HIF-1α protein rather than HIF-2α. (**A**,**B**) KC02-44D cells were exposed to 2% CSE for 4 h under 20% O_2_ conditions, and protein (**A**) and total RNA (**B**) were harvested. Whole-cell lysates were immunoblotted for HIF-2α, HIF-1β, and β-actin (**A**), and total RNA was analyzed by semi-quantitative RT-PCR for HIF1A, EPAS1, and ARNT (**B**). (**C–E**) KC02-44D cells were transfected with the HIF-1-dependent reporter gene p5HRE-luc encoding firefly luciferase (**C**), pGL3/ODD-Luc (**D**), and pGL3/HIF-1α 5′FS-Luc (**E**), followed by treatment with the indicated doses of CSE for 6 h. The luciferase activity was normalized to the value obtained from untreated cells in order to obtain relative light units. Data represent the mean ± SD of three independent transfections. * *p* < 0.05 vs. control.

We then evaluated HIF-2α protein accumulation in KC02-44D cells. In contrast to HIF-1α, the CSE treatment did not induce HIF-2α protein accumulation in KC02-44D cells (Figure 5A), and neither 2% CSE nor 1% O_2_ treatment increased the HIF1A mRNA level according to the semi-quantitative RT-PCR (Figure 5B). Additionally, neither EPAS1 nor ARNT mRNA levels were significantly affected by the 2% CSE treatment (Figure 5B). We then investigated the effect of CSE on HIF-1 activity using a hypoxia-responsive element (HRE)-luciferase reporter construct, finding that exposure to CSE or 1% O_2_ promoted HRE-dependent gene expression (Figure 5C). Since the primary mechanism leading to HIF-1α accumulation during hypoxia involves stabilization of HIF-α proteins, we examined whether CSE affects the intracellular stability of HIF-1 protein using the oxygen-dependent degradation (ODD) domain of HIF-1α fused to the luciferase protein (Figure 4D). Exposure to 1% O_2_ induced the luciferase activity, indicating that 1% O_2_ stabilized the ODD peptide. Moreover, the CSE treatment enhanced the induction of luciferase activity under 20% O_2_ conditions in a dose-dependent manner. We examined the promoter activity of HIF-1α gene using a reporter system with the 5’ flanking sequence from the HIF-1α gene fused to luciferase. Exposure to 1% O_2_ induced the luciferase activity as compared with that observed in the control (20% O_2_ conditions). Furthermore, the 2% CSE treatment did not affect the HIF-1α promoter activity under 20% O_2_ conditions (Figure 5E). These results indicated that CSE stabilized HIF-1α rather than increased its transcriptional activity.

### 3.5. Critical Involvement of ROS in HIF-1 Activation

**Figure 6:**
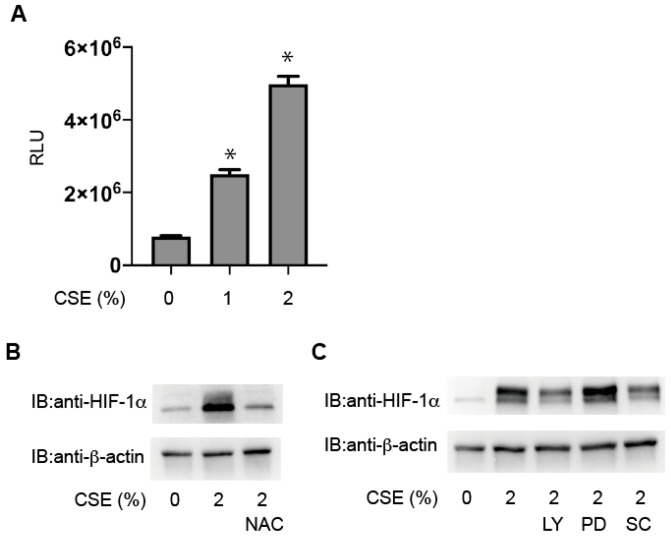
Critical involvement of reactive oxygen species (ROS) in HIF-1α activation. (**A**) KC02-44D cells exposed to 1% or 2% CSE for 4 h and ROS were assayed as described in the Materials and Methods. Data represent the mean ± SD of three independent experiments. * *p* < 0.05 vs. control. (**B**,**C**) KC02-44D cells were exposed to 2% CSE for 4 h under 20% O_2_ conditions with or without 10 mM NAC (**B**) or the indicated kinase inhibitors (**C**), and whole-cell lysates were immunoblotted for HIF-1α and β-actin. LY: LY294002; PD: PD98059; SC: SC-514.

We found that the CSE treatment increased ROS levels in KC02-44D cells (Figure 6A). To investigate the role of ROS, we examined the effect of a potent antioxidant (N-acetylcysteine (NAC)) on CSE-induced HIF-1 activation. The results showed that 40 mM NAC suppressed HIF-1α protein accumulation in CSE-treated KC02-44D cells (Figure 6B). CSE is reportedly a strong activator of phosphoinositide 3-kinase (PI3K) and mitogen-activated protein kinases (MAPKs) and previous studies suggest that PI3K and MAPK signaling is involved in HIF-1 activation [20]. To investigate whether the HIF-1α protein level is affected by PI3K or MAPK signaling in vitro, we examined the effects of the kinase inhibitors LY294002, PD98059, and SC-514. The results showed that treatment with LY294002 and SC-514, respectively, suppressed CSE-mediated HIF-1α levels (Figure 6C).

## 4. Discussion

In this study, we demonstrated that CSE induced the activation of HIF-1α in ESCs. CS toxicity tests are commonly used in animal and cell experiments, and CSE is widely employed for in vitro models [40]. Previous studies report that 1% CSE can be derived from fewer than two packs of cigarettes per day [25,26], therefore, we used 0.5 to 4% CSE for this study.

An immortalized human ESC line, KC02-44D, by introducing the hTERT gene into primary ESCs showed characteristics such as decidualization capacity and responses to IL-1β similar to the primary human ESCs [24]. It is reported that the KC02-44D cell line is useful to investigate the mechanisms of normal and pathological human reproductive processes. We found that CSE produced ROS in immortalized KC02-44D cells, although the precise mechanism remains unclear. Mitochondria and NADPH oxidases have been implicated as major sites of ROS generation in response to chronic exposure to smoke. Mitochondria are major generators of superoxide, predominantly at complexes I and III [41]. In endothelial cells, a study has shown that thiol-reactive stable compounds in CSE activate NADPH oxidase to produce ROS [42,43]. It is reported that tobacco produces ROS through inhibition of the mitochondrial electron transfer system [44,45]. These mechanisms might explain the production of ROS observed in the human endometrium. Moreover, stabilization assays using ODD-luciferase proteins revealed that CSE promoted HIF-1α protein stabilization, the mechanism of which differed between primary cultured cells and KC02-44D cells. Furthermore, the HIF-1α level was selectively upregulated in primary cultured cells as compared with the HIF-2α level, and CSE-generated ROS upregulated intracellular levels of HIF-1α in an ROS-dependent manner, consistent with previous reports showing that some effects of cigarettes are ROS-dependent [20]. A previous study indicated that ROS inhibits the HIF-1α prolyl hydroxylation enzyme [46,47], which is consistent with reports that the HIF-1α hydroxylase is redox regulated, and that H_2_O_2_ also results in the activation of HIF-1.

We used the RNA-Seq analysis in this study to investigate the effect of tobacco on comprehensive gene expression. Intriguingly, the CSE treatment was found to drive the expression of a cluster of hypoxia-induced genes in endometrial stromal cells, a finding that strongly supports the observation that CSE causes the induction of HIF-1α and HIF-2α protein expression. On the other hand, the difference between 1% O_2_-driven and CSE-driven gene changes should not be overlooked. The 1% O_2_ treatment is mainly characterized by gene clusters related to aerobic to anaerobic metabolic reprogramming, while the CSE treatment is characterized by the enrichment of genes related to cell death. Therefore, in addition to the RT-PCR analysis, RNA-Seq revealed that CSE activates HIFs through induction of HIF-1α protein expression. Another important point supported by the RNA-Seq analysis is that the effect of CSE on HIF-1α and HIF-2α is selective. The CSE treatment promotes the induction of HIF-1α protein but has a weak effect on HIF-2α protein. Based on the database of literature-curated human TF-target interactions, TRRUST (transcriptional regulatory relationships unraveled by sentence-based text-mining) we analyzed RNA-Seq results in relation to transcription factors. The results clearly indicate that HIF-1 contributes more significantly compared to HIF-2 in the CSE-induced gene expression.

We previously showed that hypoxia or CoCl_2_, a HIF-PH inhibitor, induces *VEGF* expression in human ESC while suppressing *SDF-1* (encoding C-X-C motif chemokine ligand 12) [21]. In the present study, the RNA-Seq analysis revealed that hypoxia but not CSE strongly suppressed CXCL12 levels in KC02-44D cells, and that CSE induced the expression of not only hypoxia-responsive genes but also those related to cell death. These findings indicated commonalities between CSE and hypoxic stimulation, whereas each induces distinct changes in gene expression.

A previous report demonstrated the significance of HIF-1 and HIF-2 in the uterus [16]. They generated HIF-1α-deficient (HIF1A conditional knockout) and HIF-2α-deficient (EPAS1 conditional knockout) mice and examined the pregnancy phenotype associated with HIF-2α deficiency. The findings indicated the selective importance of HIF-2α rather than HIF-1α, and that stromal HIF-2α allows trophoblast invasion through the detachment of the luminal epithelium and activation of an embryonic survival signal. A study indicates that the cyclic activation or inactivation of HIF-1 is essential for the maintenance of the endometrial environment during menstruation [48]. The sustained HIF-1 activation by smoking can affect endometrial repair during menstruation. An important point of view is that HIFs are not only activated and regulated by the change of oxygen environment, but also that the HIF activity modulates the oxygen environment by altering the mode of glucose metabolism, angiogenesis, and mitochondrial function [49,50]. We demonstrated that the activation of HIF-1 and HIF-2 is dysregulated by tobacco, which significantly changes the intrauterine environment. The dysregulation can disrupt the normal intrauterine environment, which can lead to implantation and subsequent failure of placental maturation, which may predispose to pregnancy hypertension [51,52,53].

This study has several limitations. First, we established the CSE concentration at 2% based on those used in several previous studies [54,55,56]. However, no previous study has accurately measured the concentration of tobacco components to which the uterus is exposed. CS contains >4000 chemical constituents, including high concentrations of oxidants [20,57]. A previous study demonstrated that acrolein and nicotine are active in inducing HIF-1α activation in alveolar epithelium-derived cell lines [20]. Isolation and characterization of these compounds might help clarify why and how CSE causes concentration-dependent differential gene expression and potentially promotes the development of therapeutic strategies.

## 5. Conclusions

In this study, we demonstrated that CSE reversibly activates HIFs in a ROS-dependent manner in immortalized endometrial stromal cells, as well as in primary cultured endometrial stromal cells and that ROS generated by CSE play a critical role in the activation. The results of RNA-Seq analysis using next-generation sequencing showed that CSE activates the hypoxia-inducible gene expression pathway and that HIF-1 is selectively involved in its induction compared to HIF-2. In the future, we will study the effect of CSE on endometrial stromal decidualization and the effect of CSE-HIF on fertility and embryo implantation and the subsequent course of pregnancy. Moreover, our results support epidemiological studies that cigarette smoke has an adverse effect on the reproductive outcome.

## Figures and Tables

**Table 1:** Primer sequences used for real-time polymerase chain reaction (PCR).

Target	Gene Symbol	Forward Primer 5′–3′	Reverse Primer 5′–3′
VEGF	VEGFA	CGAAACCATGAACTTTCTGC	CCTCAGTGGGCACACACTCC
GLUT1	SLC2A1	TCCACGAGCATCTTCGAGA	ATACTGGAAGCACATGCCC
HIF-1a	HIF1A	ACACACAGAAATGGCCTTGTGA	CCTGTGCAGTGCAATACCTTC
HIF-2a	EPAS1	ATGGGACTTACACAGGTGGAG	GCTCTGTGGACATGTCTTTGC
HIF-1b	ARNT	TGTTGGCTACCAGCCACAGGAACT	ACCGGAACCGGAACATGACAGA
EF1α	EEF1A1	TCTGGTTGGAATGGTGACAACATGC	AGAGCTTCACTCAAAGCTTCATGG

## Data Availability

The data presented in this study are openly available in the DNA Data Bank of Japan Sequence Read Archive (https://www.ddbj.nig.ac.jp/dra/index-e.html; accession nos. DRR228416–DRR228423).

## Abbreviations

CSE	cigarette smoke extract
HIF	hypoxia-inducible factor
ESC	endometrial stromal cell
ROS	reactive oxygen species
GSEA	gene set enrichment analysis

## Appendix A

**Table A1:** Key Resources Table.

Reagents	Source	Identifier
2-Amino-2-hydroxymethyl-1,3-propanediol	Wako	207-06275
7.5% Mini-PROTEAN^®^ TGX^TM^ Precast Protein Gels	BIO-RAD	4561023
Anti-rabbit IgG (H + L), F(ab’)2 Fragment (Alexa Fluor^®^ 488 Conjugate)	Cell Signaling TECHNOLOGY	4412
Anti-β-Actin antibody	Sigma-Aldrich	A5316
Antibiotic-Antimycotic (100X)	Gibco	5240-062
Blocking One	Nacalai Tesque	03953-95
Bovine serum albumin	Wako	017-23294
Cell Counting Kit-8	Dojindo	CK04
DEXTRAN	Sigma-Aldrich	D4751
Dithiothreitol (DTT)	BIO-RAD	161-0610
DMEM/F-12, HEPES, no phenol red	Gibco	11039-021
DMEM/F12, Phenol Red	Gibco	11330-032
ECL^TM^ Prime Western Blotting Detection Reagents	GE Healthcare	RPN2232
Ethanol (99.5)	Wako	057-00456
fetal bovine serum	Sigma-Aldrich	172012
Formaldehyde	Polysciences	18814
FuGENE^®^ HD Transfection Reagent	Promega	E2311
GlutaMAX™ Supplement	Gibco	35050-061
Glycine	Wako	077-00735
HIF-1β/ARNT(D28F3)XP rabbit monoclonal antibody	Cell Signaling TECHNOLOGY	5537
HIF-2 alpha/EPAS1 Antibody	Novus Biologicals	NB100-122
Hydrochloric Acid	Wako	084-05425
LY294002	Wako	129-04861
N-Acetyl-L-cysteine, ROS inhibitor	abcam	143032
M-PER™ Mammalian Protein Extraction Reagent	Thermo Fisher Scientific	78501
Mouse IgG HRP Linked Whole Ab	GE Healthcare	NA931
normal rabbit IgG	SANTA CRUZ BIOTECHNOLOGY	sc-2027
Opti-MEM™ I Reduced Serum Medium	Gibco	31985-062
ONE-Glo™ Luciferase Assay System	Promega	E6120
PBS, pH 7.4	Gibco	10010-023
PD98059	Wako	169-19211
Pierce™ BCA Protein Assay Kit	Thermo Fisher Scientific	23225
Polyoxyethylene(20) Sorbitan Monolaurate	Wako	167-11515
Progesterone receptor Antibody	SANTA CRUZ BIOTECHNOLOGY	sc-166169
Protease inhibitor cocktail	Calbiochem	539134
Purified Mouse Anti-Human HIF-1α	BD Transduction Laboratories^TM^	610958
ReverTra Ace^®^ qPCR RT Master Mix with gDNA Remover	TOYOBO	FSQ-301
RNeasy Mini Kit	Qiagen	74106
ROS-Glo™ H_2_O_2_ Assay	Promega	G8820
Running Buffer Solution fro SDS-PAGE	Nacalai Tesque	30329-61
Tris Bufferd Saline with Tween^®^ 20 (TBS-T) Tablets, pH 7.6	TaKaRa	79142
THUNDERBIRD^®^ SYBR qPCR Mix	TOYOBO	QPS-201
Trans-Blot^®^ Turbo™ Mini PVDF Transfer Packs	BIO-RAD	1704156
Trypsin-EDTA (0.25%)	Gibco	25200-056
SC-514	Sigma	SML0557-5MG
X-tremeGENE HP DNA Transfection Reagent	Roche	6366236001
β-Mercaptoethanol	Wako	131-14572

## References

[1] Jha P., Peto R. (2014). Global effects of smoking, of quitting, and of taxing tobacco. N. Engl. J. Med..

[2] Shiverick K.T., Salafia C. (1999). Cigarette smoking and pregnancy I: Ovarian, uterine and placental effects. Placenta.

[3] Orzabal M.R., Lunde-Young E.R., Ramirez J.I., Howe S.Y.F., Naik V.D., Lee J., Heaps C.L., Threadgill D.W., Ramadoss J. (2019). Chronic exposure to e-cig aerosols during early development causes vascular dysfunction and offspring growth deficits. Transl. Res..

[4] Sugawara Y., Tsuji I., Mizoue T., Inoue M., Sawada N., Matsuo K., Ito H., Naito M., Nagata C., Kitamura Y. (2019). Cigarette smoking and cervical cancer risk: An evaluation based on a systematic review and meta-analysis among Japanese women. Jpn. J. Clin. Oncol..

[5] Zhou Y., Jorgensen E.M., Gan Y., Taylor H.S. (2011). Cigarette smoke increases progesterone receptor and homeobox A10 expression in human endometrium and endometrial cells: A potential role in the decreased prevalence of endometrial pathology in smokers. Biol. Reprod..

[6] Nakamoto T., Yasuda K., Yasuhara M., Nakajima T., Mizokami T., Okada H., Kanzaki H. (2006). Cigarette smoke extract enhances oxytocin-induced rhythmic contractions of rat and human preterm myometrium. Reproduction.

[7] Soares S.R., Simon C., Remohi J., Pellicer A. (2007). Cigarette smoking affects uterine receptiveness. Hum. Reprod..

[8] Tsutsumi R., Hiroi H., Momoeda M., Hosokawa Y., Nakazawa F., Yano T., Tsutsumi O., Taketani Y. (2009). Induction of early decidualization by cadmium, a major contaminant of cigarette smoke. Fertil. Steril..

[9] Dechanet C., Anahory T., Mathieu Daude J.C., Quantin X., Reyftmann L., Hamamah S., Hedon B., Dechaud H. (2011). Effects of cigarette smoking on reproduction. Hum. Reprod. Update.

[10] Esposito E.R., Horn K.H., Greene R.M., Pisano M.M. (2008). An animal model of cigarette smoke-induced in utero growth retardation. Toxicology.

[11] Salafia C., Shiverick K. (1999). Cigarette smoking and pregnancy II: Vascular effects. Placenta.

[12] Rodesch F., Simon P., Donner C., Jauniaux E. (1992). Oxygen measurements in endometrial and trophoblastic tissues during early pregnancy. Obstet. Gynecol..

[13] Gassmann M., Fandrey J., Bichet S., Wartenberg M., Marti H.H., Bauer C., Wenger R.H., Acker H. (1996). Oxygen supply and oxygen-dependent gene expression in differentiating embryonic stem cells. Proc. Natl. Acad. Sci. USA.

[14] Hirota K. (2020). Basic Biology of Hypoxic Responses Mediated by the Transcription Factor HIFs and Its Implication for Medicine. Biomedicines.

[15] Daikoku T., Matsumoto H., Gupta R.A., Das S.K., Gassmann M., DuBois R.N., Dey S.K. (2003). Expression of hypoxia-inducible factors in the peri-implantation mouse uterus is regulated in a cell-specific and ovarian steroid hormone-dependent manner. Evidence for differential function of HIFs during early pregnancy. J. Biol. Chem..

[16] Matsumoto L., Hirota Y., Saito-Fujita T., Takeda N., Tanaka T., Hiraoka T., Akaeda S., Fujita H., Shimizu-Hirota R., Igaue S. (2018). HIF2alpha in the uterine stroma permits embryo invasion and luminal epithelium detachment. J. Clin. Investig..

[17] Majmundar A.J., Wong W.J., Simon M.C. (2010). Hypoxia-inducible factors and the response to hypoxic stress. Mol. Cell.

[18] Ryan H.E., Lo J., Johnson R.S. (1998). HIF-1 alpha is required for solid tumor formation and embryonic vascularization. EMBO J..

[19] McGettrick A.F., O’Neill L.A.J. (2020). The Role of HIF in Immunity and Inflammation. Cell Metab..

[20] Daijo H., Hoshino Y., Kai S., Suzuki K., Nishi K., Matsuo Y., Harada H., Hirota K. (2016). Cigarette smoke reversibly activates hypoxia-inducible factor 1 in a reactive oxygen species-dependent manner. Sci. Rep..

[21] Tsuzuki T., Okada H., Cho H., Shimoi K., Miyashiro H., Yasuda K., Kanzaki H. (2013). Divergent regulation of angiopoietin-1, angiopoietin-2, and vascular endothelial growth factor by hypoxia and female sex steroids in human endometrial stromal cells. Eur. J. Obstet. Gynecol. Reprod. Biol..

[22] Tsuzuki T., Okada H., Cho H., Tsuji S., Nishigaki A., Yasuda K., Kanzaki H. (2012). Hypoxic stress simultaneously stimulates vascular endothelial growth factor via hypoxia-inducible factor-1alpha and inhibits stromal cell-derived factor-1 in human endometrial stromal cells. Hum. Reprod..

[23] Okada H., Nakajima T., Sanezumi M., Ikuta A., Yasuda K., Kanzaki H. (2000). Progesterone enhances interleukin-15 production in human endometrial stromal cells in vitro. J. Clin. Endocrinol. Metab..

[24] Yuhki M., Kajitani T., Mizuno T., Aoki Y., Maruyama T. (2011). Establishment of an immortalized human endometrial stromal cell line with functional responses to ovarian stimuli. Reprod. Biol. Endocrinol..

[25] Rius C., Company C., Piqueras L., Cerda-Nicolas J.M., Gonzalez C., Servera E., Ludwig A., Morcillo E.J., Sanz M.J. (2013). Critical role of fractalkine (CX3CL1) in cigarette smoke-induced mononuclear cell adhesion to the arterial endothelium. Thorax.

[26] Su Y., Han W., Giraldo C., De Li Y., Block E.R. (1998). Effect of cigarette smoke extract on nitric oxide synthase in pulmonary artery endothelial cells. Am. J. Respir. Cell Mol. Biol..

[27] Wakamatsu T., Tanaka T., Oda S., Nishi K., Harada H., Daijo H., Takabuchi S., Kai S., Fukuda K., Hirota K. (2009). The intravenous anesthetics barbiturates inhibit hypoxia-inducible factor 1 activation. Eur. J. Pharmacol..

[28] Kobayashi M., Morinibu A., Koyasu S., Goto Y., Hiraoka M., Harada H. (2017). A circadian clock gene, PER2, activates HIF-1 as an effector molecule for recruitment of HIF-1alpha to promoter regions of its downstream genes. FEBS J..

[29] Zeng L., Morinibu A., Kobayashi M., Zhu Y., Wang X., Goto Y., Yeom C.J., Zhao T., Hirota K., Shinomiya K. (2015). Aberrant IDH3alpha expression promotes malignant tumor growth by inducing HIF-1-mediated metabolic reprogramming and angiogenesis. Oncogene.

[30] Kakita-Kobayashi M., Murata H., Nishigaki A., Hashimoto Y., Komiya S., Tsubokura H., Kido T., Kida N., Tsuzuki-Nakao T., Matsuo Y. (2020). Thyroid Hormone Facilitates in vitro Decidualization of Human Endometrial Stromal Cells via Thyroid Hormone Receptors. Endocrinology.

[31] Sumi C., Matsuo Y., Kusunoki M., Shoji T., Uba T., Iwai T., Bono H., Hirota K. (2019). Cancerous phenotypes associated with hypoxia-inducible factors are not influenced by the volatile anesthetic isoflurane in renal cell carcinoma. PLoS ONE.

[32] Hiraoka Y., Yamada K., Kawasaki Y., Hirose H., Matsumoto K., Ishikawa K., Yasumizu Y. (2019). Ikra: RNAseq pipeline centered on Salmon. Zenodo.

[33] Patro R., Duggal G., Love M.I., Irizarry R.A., Kingsford C. (2017). Salmon provides fast and bias-aware quantification of transcript expression. Nat. Methods.

[34] Krueger F. Trim Galore. https://www.bioinformatics.babraham.ac.uk/projects/trim_galore/.

[35] Martin M. (2011). Cutadapt removes adapter sequences from high-throughput sequencing reads. EMBnet J..

[36] Ge S.X., Son E.W., Yao R. (2018). iDEP: An integrated web application for differential expression and pathway analysis of RNA-Seq data. BMC Bioinform..

[37] Zhou Y., Zhou B., Pache L., Chang M., Khodabakhshi A.H., Tanaseichuk O., Benner C., Chanda S.K. (2019). Metascape provides a biologist-oriented resource for the analysis of systems-level datasets. Nat. Commun..

[38] Han H., Cho J.W., Lee S., Yun A., Kim H., Bae D., Yang S., Kim C.Y., Lee M., Kim E. (2018). TRRUST v2: An expanded reference database of human and mouse transcriptional regulatory interactions. Nucleic Acids Res..

[39] Han H., Shim H., Shin D., Shim J.E., Ko Y., Shin J., Kim H., Cho A., Kim E., Lee T. (2015). TRRUST: A reference database of human transcriptional regulatory interactions. Sci. Rep..

[40] Kim S.M., Hwang K.A., Go R.E., Sung J.H., Choi D.W., Choi K.C. (2019). Exposure to cigarette smoke via respiratory system may induce abnormal alterations of reproductive organs in female diabetic rats. Environ. Toxicol..

[41] Han S., Chandel N.S. (2019). There Is No Smoke without Mitochondria. Am. J. Respir. Cell Mol. Biol..

[42] Jaimes E.A., DeMaster E.G., Tian R.X., Raij L. (2004). Stable compounds of cigarette smoke induce endothelial superoxide anion production via NADPH oxidase activation. Arterioscler. Thromb. Vasc. Biol..

[43] Cui Z., Han Z., Li Z., Hu H., Patel J.M., Antony V., Block E.R., Su Y. (2005). Involvement of calpain-calpastatin in cigarette smoke-induced inhibition of lung endothelial nitric oxide synthase. Am. J. Respir. Cell Mol. Biol..

[44] Biswas S., Das H., Das U., Sengupta A., Dey Sharma R., Biswas S.C., Dey S. (2020). Smokeless tobacco induces toxicity and apoptosis in neuronal cells: A mechanistic evaluation. Free Radic. Res..

[45] Perez-Rial S., Barreiro E., Fernandez-Acenero M.J., Fernandez-Valle M.E., Gonzalez-Mangado N., Peces-Barba G. (2020). Early detection of skeletal muscle bioenergetic deficit by magnetic resonance spectroscopy in cigarette smoke-exposed mice. PLoS ONE.

[46] Semenza G.L., Prabhakar N.R. (2012). The role of hypoxia-inducible factors in oxygen sensing by the carotid body. Adv. Exp. Med. Biol..

[47] Semenza G.L., Prabhakar N.R. (2018). The role of hypoxia-inducible factors in carotid body (patho) physiology. J. Physiol..

[48] Maybin J.A., Murray A.A., Saunders P.T.K., Hirani N., Carmeliet P., Critchley H.O.D. (2018). Hypoxia and hypoxia inducible factor-1alpha are required for normal endometrial repair during menstruation. Nat. Commun..

[49] Semenza G.L. (2007). Life with oxygen. Science.

[50] Semenza G.L. (2000). HIF-1 and human disease: One highly involved factor. Genes Dev..

[51] Zhan L., Wang W., Zhang Y., Song E., Fan Y., Wei B. (2016). Hypoxia-inducible factor-1alpha: A promising therapeutic target in endometriosis. Biochimie.

[52] Sava R.I., March K.L., Pepine C.J. (2018). Hypertension in pregnancy: Taking cues from pathophysiology for clinical practice. Clin. Cardiol..

[53] Patel J., Landers K., Mortimer R.H., Richard K. (2010). Regulation of hypoxia inducible factors (HIF) in hypoxia and normoxia during placental development. Placenta.

[54] Lee H.M., Choi K.C. (2018). Cigarette smoke extract and isoprene resulted in the induction of apoptosis and autophagy in human placenta choriocarcinoma JEG-3 cells. Environ. Toxicol..

[55] Lee S.D., Lee D.S., Chun Y.G., Shim T.S., Lim C.M., Koh Y., Kim W.S., Kim D.S., Kim W.D. (2001). Cigarette smoke extract induces endothelin-1 via protein kinase C in pulmonary artery endothelial cells. Am. J. Physiol. Lung Cell. Mol. Physiol..

[56] Lee H.M., Kim C.W., Hwang K.A., Sung J.H., Lee J.K., Choi K.C. (2017). Cigarette smoke impaired maturation of ovarian follicles and normal growth of uterus inner wall of female wild-type and hypertensive rats. Reprod. Toxicol..

[57] Bernhard D., Wang X.L. (2007). Smoking, oxidative stress and cardiovascular diseases—Do anti-oxidative therapies fail?. Curr. Med. Chem..

